# A Mouse Model of Multiple System Atrophy: Bench to Bedside

**DOI:** 10.1007/s13311-022-01287-8

**Published:** 2022-08-22

**Authors:** Nadia Stefanova

**Affiliations:** Laboratory for Translational Neurodegeneration Research, Department of Neurology, Medical University of Innsbruck, Innsbruck, Austria

**Keywords:** Multiple system atrophy, Alpha-synuclein, Mouse model, Therapy, Disease mechanisms

## Abstract

Multiple system atrophy (MSA) is a rare neurodegenerative disorder with unclear etiology, currently difficult and delayed diagnosis, and rapid progression, leading to disability and lethality within 6 to 9 years after symptom onset. The neuropathology of MSA classifies the disease in the group of a-synucleinopathies together with Parkinson’s disease and other Lewy body disorders, but features specific oligodendroglial inclusions, which are pathognomonic for MSA. MSA has no efficient therapy to date. Development of experimental models is crucial to elucidate the disease mechanisms in progression and to provide a tool for preclinical screening of putative therapies for MSA. In vitro and in vivo models, based on selective neurotoxicity, a-synuclein oligodendroglial overexpression, and strain-specific propagation of a-synuclein fibrils, have been developed, reflecting various facets of MSA pathology. Over the years, the continuous exchange from bench to bedside and backward has been crucial for the advancing of MSA modelling, elucidating MSA pathogenic pathways, and understanding the existing translational gap to successful clinical trials in MSA. The review discusses specifically advantages and limitations of the PLP-a-syn mouse model of MSA, which recapitulates motor and non-motor features of the human disease with underlying striatonigral degeneration, degeneration of autonomic centers, and sensitized olivopontocerebellar system, strikingly mirroring human MSA pathology.

**Supplementary Information:**

The online version contains supplementary material available at 10.1007/s13311-022-01287-8.

## Multiple System Atrophy — Introduction

The term multiple system atrophy (MSA) was introduced in 1969 by Oppenheimer and Graham [1]. They observed overlapping clinical presentations in the syndromes of sporadic olivopontocerebellar atrophy (OPCA), striatonigral degeneration (SND), and Shy-Drager syndrome and therefore suggested the unifying diagnosis of MSA. The accuracy of this suggestion was confirmed 20 years later by the neuropathological observation of argyrophilic inclusion bodies with “tubular structure” in the oligodendrocytes of patients with different combinations of MSA syndromes. These oligodendroglial aggregates were named glial cytoplasmic inclusions (GCIs) [2]. Another 9 years were needed to identify filamentous alpha-synuclein (a-syn) as a component of GCIs linking MSA with Parkinson’s disease (PD) and dementia with Lewy bodies (DLB) within the group of a-synucleinopathies [3–5].

## Epidemiological Features and Natural History of MSA

MSA is a rare, rapidly progressive neurodegenerative disorder with the profile of an orphan disease. The usual symptom onset is in the fifth decade of life. Its incidence ranges between 0.1 and 2.4/100,000 per year increasing with age, and the estimated prevalence is 4.4/100,000 [6, 7]. Men and women are similarly affected. The disease duration after first diagnosis is 7.9 ± 2.8 years, i.e., much shorter than in PD [8]. The spectrum of symptoms in MSA includes parkinsonism, ataxia, autonomic dysfunction, and pyramidal signs in various combinations [9]. The studies on the natural history of the disease indicate that usually, non-motor symptoms are the first to be reported. These may include orthostatic hypotension, urogenital dysfunction, sleep, and respiratory disorder (stridor). The actual clinical diagnosis of MSA according to the current criteria is possible only after the onset of motor symptoms, which, based on the predominant syndrome, define the Parkinsonian variant (MSA-P) or the cerebellar variant (MSA-C) [10]. The clinical diagnosis can be set with different degrees of certainty (possible or probable), but the final diagnosis is currently possible only postmortem with the demonstration of GCIs in the brain. The diagnostic accuracy is often reduced by the overlapping symptoms with other disorders like PD, DLB, or progressive supranuclear palsy (PSP) [11]. The clinical decline of MSA patients is very rapid, and they may reach milestones of disability within a short period of about 5 years after the first diagnosis [8]. The devastating character of MSA is determined not only by its rash progression but also by the lack of efficient therapy. The symptomatic treatments have usually limited benefit and are unable to stop the progression of the degeneration [9].

In summary, MSA presents a serious medical and social problem with its delayed diagnosis according to the current criteria, low diagnostic accuracy, rapid progression, and early disability of the patients parallel to the lack of efficient therapy. For all these reasons, it is of paramount importance to understand the disease mechanisms and define molecular targets that may support an improved early diagnosis as well as serve as the key towards disease modification in MSA.

## The Neuropathology of MSA — Milestones and Recent Discoveries

One of the main sources to get a glimpse into the disease mechanisms is usually the neuropathological examination of the brain. The neuropathological milestones of MSA include widespread pathognomonic a-syn-positive GCIs, selective neurodegeneration with SND and OPCA of various severity and combinations, and degeneration of autonomic CNS centers in the brainstem and spinal cord. In the postmortem brain, gliosis, myelin changes, and demyelination usually accompany the neurodegeneration [12–17]. Neuroinflammatory signatures in the MSA brain include microglial upregulation of toll-like receptor 4 (TLR4) [18], myeloperoxidase (MPO) [19], and inflammasome-related proteins like NLRP3, ASC, and caspase 1 [20]. In addition, the pro-inflammatory cytokines TNF-α, IL-1β, and Il-6 are found increased in the cerebrospinal fluid (CSF) of MSA patients [21]. Therefore, neuroinflammatory changes linked to microglial activation have been suggested as a possible player in MSA pathogenesis [22]. The myelin dysfunction with possible early relocation and accumulation of p25a/TPPP from the myelin sheaths to the oligodendroglial soma has indicated a possible primary oligodendrogliopathy [17, 23], which may be an early pathogenic event in the disease cascade. Finally, a specificity of a-synucleinopathy has been described at cellular and molecular level in MSA as compared to Lewy body (LB) disorders. A-syn in GCIs is rich in post-translational modifications like phosphorylation at Ser129 [24] and widespread nitration [25]. The initially shown disease-specific widespread ectopic aggregation of a-syn in oligodendrocytes in MSA is accompanied by neuronal cytoplasmic and nuclear a-syn inclusions, which structurally differ from LBs [26–29]. Recent findings have suggested a different structure of the a-syn fibrils in MSA with a different seeding profile as compared to other a-synucleinopathies [30, 31]. It is unclear yet whether the disease-specific a-syn strains are causative for the different a-synucleinopathies or rather represent a secondary event of specific misfolding within a different pathogenic environment. It is known that a-syn fibrils are not the only constituent of GCIs and the structure of these pathological aggregates includes a large number of other components [32]. Neuropathological analysis has suggested the disruption of the ubiquitin–proteasome system (UPS) and the autophagy-lysosomal pathway (ALP) in MSA [33–35], but it remains unclear whether these defects have a causative role in GCI formation or rather represent a consequence of the effects of misfolded a-syn on the function of UPS and ALP. Finally, it is still under debate whether the inclusion pathology in MSA plays a detrimental role in the disease pathogenesis or represents a rescue mechanism of the cells and acts as a “trash bin” for the misfolded proteins accumulating in the cell. The origin of a-syn in oligodendrocytes of MSA is largely uncertain. Earlier studies have claimed that a-syn is a neuronal protein, which is not expressed in mature oligodendroglia [36]. However, laser dissection of oligodendroglia from MSA and control brains has suggested that MSA oligodendrocytes show a tendency to express more SNCA mRNA than control oligodendrocytes [37], supporting a possible oligodendroglial a-synucleinopathy. Oligodendroglial progenitor cells (OPCs) are known to express SNCA mRNA, but maturation to oligodendrocytes is associated with physiological decline of a-syn expression [38]. A postmortem analysis in MSA patients has proposed an increased number of striatal OPCs [39], which may indicate a dysfunctional maturation of the oligodendroglial lineage in MSA. Although the density of OPCs has been identified increased in the white matter of the MSA brain, it has been linked to demyelination, but not to accumulation of a-syn in OPCs [40].

The findings in minimal change MSA cases, in which the disease is characterized by widespread GCIs, restricted neuronal loss, and short duration, have suggested that a-syn-associated oligodendroglial pathology may lead to neuronal dysfunction sufficient to cause clinical symptoms before overt neuronal loss in MSA [41–43].

Finally, the neuropathological examination of peripheral and autonomic nerves in MSA has evidenced the presence of phosphorylated S129 a-syn and a-syn oligomers with nerve fiber degeneration in the skin [44]. Importantly, Schwann cells in cranial and spinal nerves, spinal and sympathetic ganglia, but only rarely in visceral nerves have been shown to form filamentous a-syn inclusions of phosphorylated a-syn in MSA [45]. To that, myenteric neurons in MSA have been reported to present with shrinkage of the soma without phosphorylated a-syn accumulation [46]. Although the number of studies on the peripheral a-synucleinopathy in MSA is limited, the notion is that Schwann cell synucleinopathy may precede the nerve dysfunction similar to the a-syn oligodendroglial pathology in the CNS.

## Evidence on the Cause of MSA so far — the Possible Interplay of Genetics and Environment

So far, the cause of MSA is unknown. No mutations have been identified in the coding region of SNCA [47]. COQ2 mutations, linked to mitochondrial dysfunction, have been linked to family cases of MSA in the Japanese population, but not in other cohorts [48, 49]. Genome wide association studies (GWAS) identified several potentially interesting gene loci, including the FBXO47, ELOVL7, EDN1, and MAPT, but no association of SNCA and COQ2 variants with MSA [50]. Importantly, MSA and inflammatory bowel disease have been reported to share common genetics including common variants of the C7 gene supporting immune dysfunction in both disorders [51]. The current understanding is that certain genetic background may predispose to MSA. On the other hand, environmental factors associated with oxidative stress and toxicity like those linked to occupational history of farming may be more common in MSA cases as in controls [52].

In summary, MSA is a multifactorial disorder with a rapid progression and selective neuronal loss possibly mediated by a-syn pathology, oligodendroglial dysfunction, and neuroinflammatory signaling. Disease models are instrumental in understanding the contribution of each of these components in the pathogenesis of the disease and provide a testbed for novel therapeutic approaches for MSA.

## Classes of MSA Models and Their Relevance

Modelling MSA has been approached both in vitro in cell culture and in vivo in rodents and non-human primates. The early in vitro models have been mostly based on a-syn overexpression in glial cells (primary or cell lines) to study the effects of a-syn on their biology in respect to survival [53], susceptibility to oxidative stress and pro-inflammatory signals [54–56], and the role of p25a/TPPP in GCI formation [57]. Such models have been further relevant as biosensor systems to study the seeding properties of MSA-derived a-syn oligomers versus those derived from PD brains [58]. Recently, induced pluripotent stem cells (iPSCs) have been reprogrammed from somatic cells (fibroblasts or blood cells) of MSA patients and further differentiated into neurons disclosing possible mitochondrial dysfunction in MSA as compared to cells of healthy controls [59, 60]. MSA iPSC-derived neural progenitor cells (NPCs), which give rise to both neuronal and glial cells, have been found to compensate functionally the putative mitochondrial deficit at baseline as compared to cells of healthy controls. However, the MSA cellular pathology becomes apparent in the dish after exposure to very low doses of oxidative stress [61], further consolidating the idea of the multifactorial origin of MSA with a combined role of a genetic predisposition and environmental trigger.

The in vivo models of MSA have been focusing on replicating the neuropathological and symptomatic phenotype of the disease. Initial neurotoxin models tried to replicate the SND by combining selective striatal and nigral neurotoxins [62–67]. These models have been instrumental to study the pathophysiology of SND, but their major limitation has been the lack of a-syn pathology.

Recently, the finding of MSA-specific a-syn strains [30, 31, 58] and their prion-like spreading [68–70] has triggered not only significant interest in this rare disease, but has opened a new avenue for preclinical in vivo modelling based on the strain-specific spreading of a-syn [71]. However, typical GCIs, which are widely spread in the human MSA brain, are not readily seen in the rodent brain after intracerebral inoculation of a-syn fibrils. This discrepancy may be due to the different neurobiology of mice and humans, the limited experimental observation periods, a difference between the in vitro generated PFFs and the pathological a-syn fibrils in patients, or other technical issues. Similar observations have been seen when a-syn fibrils have been introduced through the external urethral sphincter or detrusor, propagating to the CNS [72]. The general notion from an increasing number of studies using a-syn spreading models confirms that healthy oligodendrocytes are not readily accumulating fibrillar a-syn in their cytoplasm and possibly a preceding oligodendroglial dysfunction is needed to trigger the GCI formation. This has been supported by a recent experiment, in which only transgenic mice with oligodendroglial a-syn overexpression which get intracerebral inoculation of a-syn polymorphs are prone to accelerating an MSA-like phenotype in a strain-dependent manner [73]. The a-syn spreading models are crucial for understanding the specific features of protein misfolding and properties in MSA versus other synucleinopathies, and thus shed light on pathogenic mechanisms involved in the progression of the disease [74]. Unfortunately, to date, these models have not been able to recapitulate convincingly MSA symptomatology with its characteristic underlying selective neurodegenerative pathology.

The third strategy to model MSA has been the overexpression of a-syn in oligodendrocytes either in constitutive or inducible transgenic mice [75–79] or by AAV targeted a-syn overexpression in the substantia nigra and striatum of mice, rats, or primates [80–83]. In all overexpression models, irrespective of the mode of overexpression, a delayed progressive neurodegeneration with variable phenotype and intensity has been identified to accompany the formation of GCI-like structures in parallel to signs of neuroinflammation. All these findings have supported the causative role of oligodendroglial a-synucleinopathy in MSA neurodegeneration. However, related to the specific overexpression approach, different patterns of selective neurodegeneration, neuroinflammation, and specific functional phenotypes have been reported.

## The PLP-a-Syn Transgenic Mouse — Progressive MSA-Like Neuropathology, Motor, and Non-motor Phenotype

The PLP-a-syn transgenic mouse is generated by overexpression of human wild-type a-syn under the proteolipid protein (PLP) promoter to drive the transgene in oligodendrocytes [75]. This genetic modification results in the progressive accumulation, oligomerization, and aggregation of a-syn in oligodendrocytes throughout the CNS replicating the GCI pathology of human MSA [75, 78]. This finding is similar to observations in other transgenic mice with constitutive overexpression of human a-syn in oligodendroglia under alternative cell-specific promoters like the myelin basic protein (MBP) or the 2′,3′-cyclic-nucleotide 3′-phosphodiesterase (CNP) promoters [76, 77]. Intriguingly, the PLP-a-syn mouse shows progressive nigral and striatal neurodegeneration, modelling SND of the MSA-P subtype [78]. Nigral neuronal loss is detectable already at 4 months of age of the PLP-a-syn mice [18], while the loss of GABAergic medium spiny neurons in the striatum is detected at 12 months of age [78]. In comparison, the MBP-a-syn mouse model has been recently suggested to represent a model of MSA-C with loss of Purkinje cells detected at 4 months of age [84]. Interestingly, the PLP-a-syn mouse shows increased vulnerability of the olivopontocerebellar system to exogenous mitochondrial stress induced by 3-nitropropionic acid [85] and proteolytic dysfunction triggered by proteasome inhibition [86], leading to OPCA in this MSA model, but never in wild-type mice. Linked to this underlying neuropathology, the PLP-a-syn mouse shows progressive motor disability becoming overt at 6 months of age including shortened stride length, elevated stride length variability, slowness, and loss of balance and coordination evidenced in beam walking and, later on, in pole climbing [78]. In addition, the PLP-a-syn mouse model presents several non-motor deficits, which replicate classical non-motor symptoms in MSA. Among those is the neurogenic bladder dysfunction with a typical detrusor-sphincter dyssynergia and increased postvoid residual urine volume, associated with early loss of parasympathetic outflow neurons in the lumbosacral intermediate columns of the spinal cord already at 2 months of age and delayed degeneration of the pontine micturition center [87]. Brainstem centers, including the locus coeruleus, the nucleus ambiguus, the laterodorsal tegmental nucleus, and the pedunculopontine tegmental nucleus, degenerate early in the individual life of PLP-a-syn mice [88, 89]. This pathology leads to cardiovascular symptoms (increased heart rate variability [88]), respiratory deficits [90], and sleep disturbances including rapid eye movement (REM) sleep without atonia [91] replicating human MSA premotor symptoms [9]. Intriguingly, the widespread GCI pathology in the PLP-a-syn mouse brain leads to strictly selective neuronal loss. For example, the olfactory bulbs show oligodendroglial a-syn accumulation without loss of tyrosine hydroxylase-positive neurons and no disturbances in the olfactory function [92], which recapitulates human MSA in contrast to the early loss of smell in PD [93, 94].

**Fig. 1:**
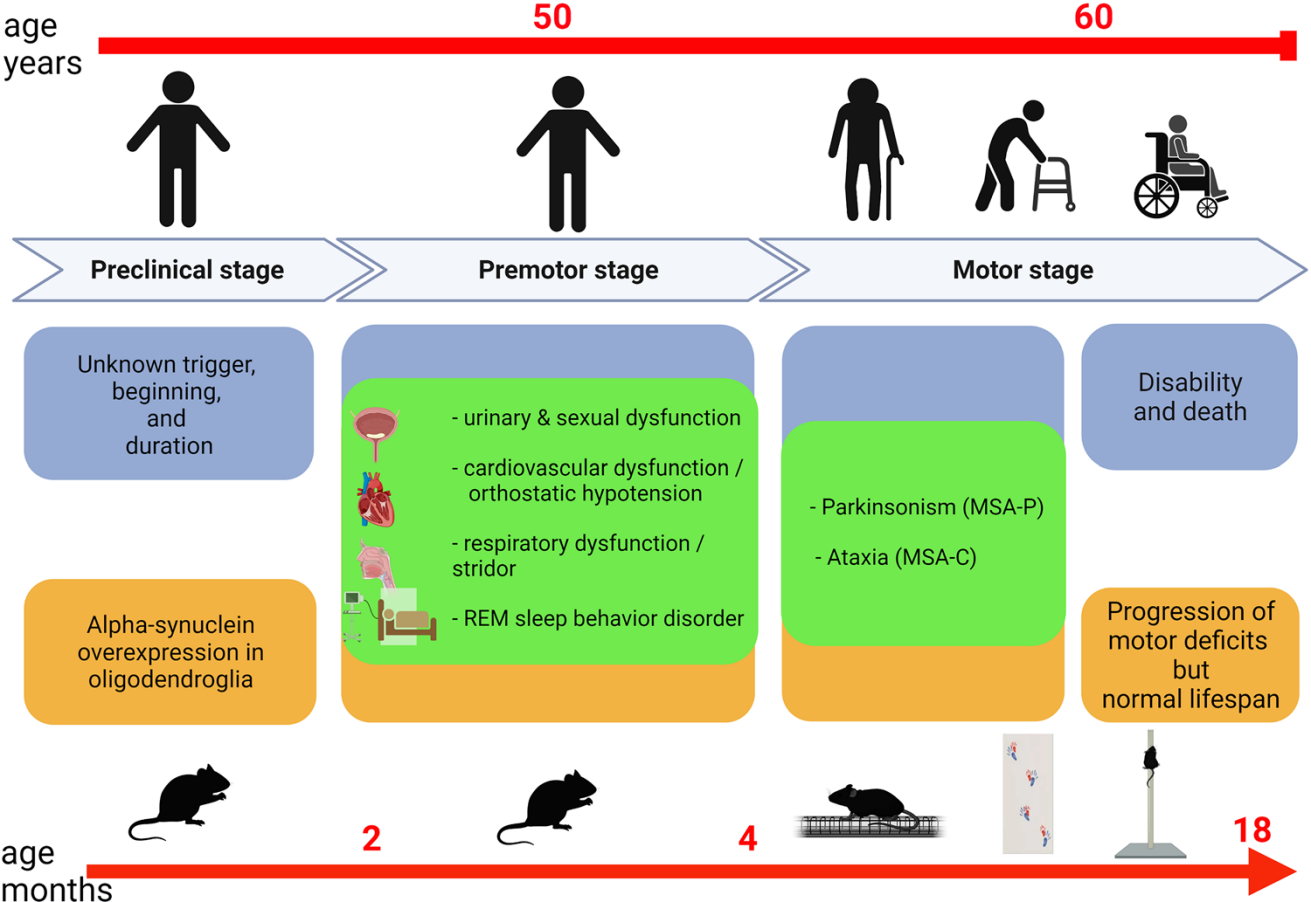
The PLP-a-syn mouse model of MSA — an excellent phenotypic replication of human MSA. Both premotor and progressive motor symptoms are replicated in the PLP-a-syn mouse comparable to the human clinical presentation (green overlaps). The limitations of the model are related to: (i) the initiation of the disease — elusive in human MSA versus a-syn overexpression in oligodendrocytes in PLP-a-syn mice; (ii) the milder phenotype and usually normal lifespan in PLP-a-syn mice versus severe disability and premature death of MSA patients; and (iii) significant differences in the normal biology between mice and humans (lifespan measured in months versus years, respectively), which may interfere with time of disease progression. Created with BioRender.com

**Fig. 2:**
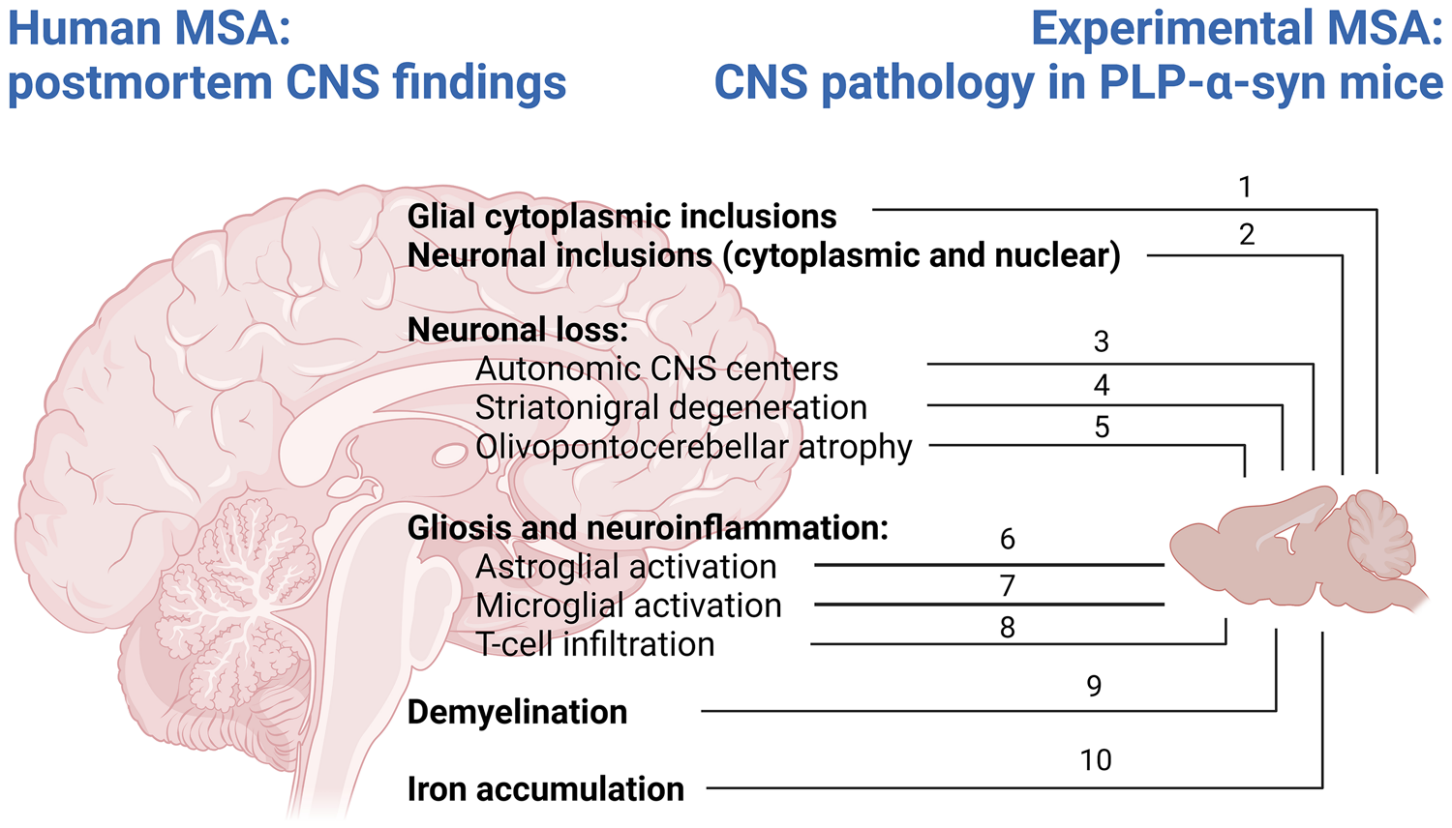
The neuropathology in PLP-a-syn mice — recapitulation of the postmortem findings in human MSA and definition of major therapeutic targets. Legend: (1) glial cytoplasmic inclusions (GCIs) are the hallmark of human MSA pathology with unknown origin, while in transgenic mice, GCIs are triggered by overexpression of human a-syn under the PLP promotor in oligodendroglia, which over time leads to high molecular weight a-syn aggregates [78]; neuronal a-syn in vesicular bodies has been observed in dopaminergic nigral neurons possibly due to cell-to-cell propagation of the oligomeric forms [78]; (3) multiple autonomic centers in the brain and spinal cord of PLP-a-syn mice show selective neuronal loss [87–90]; (4) nigral and striatal neuronal loss feature the underlying motor pathology in PLP-a-syn mice [78]; (5) olivopontocerebellar pathology can be triggered by mitochondrial or proteolytic stress in the PLP-a-syn mouse [85, 86]; (6–8) the neurodegeneration is mediated through gliosis and neuroinflammatory signaling [18, 85, 112]; (9) early signs of oligodendroglial and myelin dysfunction [95] are accelerated by proteolytic stress [86]; and (10) iron deposition in the degenerating areas can be identified [102, 105]. Created with BioRender.com

In summary, the PLP-a-syn mouse provides an excellent phenotypic replication of human MSA that proves the strong face validity of the model (Fig. 1, Fig. 2). This MSA mouse has served to study the mechanisms of MSA neurodegeneration and understand the causative role of oligodendroglial a-synucleinopathy. It recapitulates oligodendroglial a-syn-triggered SND with increased vulnerability of the olivopontocerebellar system to exogenous stressors as well as degeneration in autonomic centers. In addition, the PLP-a-syn mouse presents with progressive region-specific microglial activation triggered by the oligomeric a-syn accumulation [78] reminiscent of the microgliosis reported in postmortem analysis of MSA brains [14, 15]. The analysis of the model proposes important role of microglial activation and neuroinflammatory responses in driving the progression of the disease [18, 78]. Alternatively, demyelination is seen in end-stage MSA [15], while in the PLP-a-syn mouse, myelin dysfunction is detected without evident loss of myelin up to 18 months of age [78, 95]. Similarly, astrogliosis is not a prominent pathological feature in the progression of disease in the PLP-a-syn mouse model [78], despite the presence of astrogliosis in the postmortem MSA brains [96]. Prominent demyelination and astrogliosis may represent secondary late events in the pathogenesis of MSA and due to the limited observation time and overall duration of the disease in the PLP-a-syn model may not be detected in the mouse brain. However, demyelination and astrogliosis, but not microglial activation and SND, are reported as part of the neurodegenerative process in the MBP-a-syn mouse [76, 97], proposing that oligodendroglial a-syn overexpression in the two transgenic models may switch on different pathogenic pathways, defining different selective neurodegeneration and phenotype. This difference has not been addressed to date, but one putative explanation may be the generation of different a-syn oligomeric polymorphs, which define each of the phenotypes.

## The MSA Transgenic Mouse — a Preclinical Therapeutic Testbed for MSA with Advantages and Limitations

Preclinical therapeutic screening provides the rationale for any clinical trial. The relevance of the applied experimental model to the tested therapeutic target is crucial to ensure meaningful outcomes. All relevant current MSA models are based on the assumption that pathological a-syn is the cause of MSA neurodegeneration. On one hand, such a-syn models of MSA are advantageous when testing a-syn targeting treatment strategies, because they provide a clear-cut readout of efficacy based on a-syn pathology modulation [98–105]. Furthermore, the a-syn-based models of MSA provide the possibility to screen targeting of other relevant pathways and disease mechanisms downstream of a-syn pathology like neuroinflammation [18, 19, 78, 106, 107], neurotrophic disbalance [108, 109], epigenetic impairment [110], or demyelination [97]. On the other hand, the mechanistic replication of a-synucleinopathy in the rodent CNS may deviate from the actual trigger(s) of the human disease, as the etiology of the disease remains elusive. In combination, the limited knowledge on the initiation of MSA, the inter-species neurobiological differences between rodents and humans, and the common deviation in study design (drug dose, time of therapy initiation and relative duration, readouts, etc.) between preclinical studies and clinical trials may contribute in part to the still disappointing outcomes of clinical trials in MSA. The lack of relevant biomarkers, which may serve to monitor the biological activity of any intervention in relation to slowing disease progression in MSA patients, has been critical. The recent reports on possible progression biomarkers like neurofilament light chain [111] or neuroimaging features including advances in a-syn imaging will be crucial to provide relevant measures of target engagement in the near future.

## Conclusions and Future Directions

In summary, the models with targeted oligodendroglial overexpression of wild-type human a-syn provide a good replication of MSA-like neurodegeneration induced by oligodendroglial a-synucleinopathy. The PLP-a-syn mouse offers the most complete mechanistic recapitulation of the MSA phenotype with neurogenic bladder dysfunction, cardiovascular and respiratory deficits, REM sleep abnormalities, and progressive motor disability with underlying striatonigral degeneration and increased susceptibility of the olivopontocerebellar system to exogenous stress factors like oxidative or proteolytic stress. The limitations of the model relate to the differences in the neurobiology between mice and humans as well as the lacking information on the initiation event(s) in human MSA that makes their replication obscure in any of the current models. The PLP-a-syn mouse serves well to test the target engagement and efficacy of therapies targeting a-syn pathology and downstream pathways including neuroinflammation, disrupted neurotrophic support, and others (Fig. 2); however, its positive predictive validity has not yet been confirmed. Future development of MSA models involving human disease-specific cells may be needed to overcome the existing limitations and provide better preclinical tools for biomarker and treatment development for MSA.

## Supplementary Information

Below is the link to the electronic supplementary material.

Supplementary file1 (PDF 778 KB)

## Data Availability

Disclosure form provided by the author are available with the online version of this article.
